# Free Periosteal Flaps with Scaffold: An Overlooked Armamentarium for Maxillary and Mandibular Reconstruction

**DOI:** 10.3390/cancers13174373

**Published:** 2021-08-30

**Authors:** Remo Accorona, Luca Gazzini, Roberto Grigolato, Enrico Fazio, Letizia Nitro, Monir Abousiam, Giovanni Giorgetti, Lorenzo Pignataro, Pasquale Capaccio, Luca Calabrese

**Affiliations:** 1 Department of Otorhinolaryngology—Head and Neck Surgery, Fondazione IRCCS Ca’ Granda Ospedale Maggiore Policlinico, 20122 Milano, Italy; letizia.nitro@unimi.it (L.N.); lorenzo.pignataro@unimi.it (L.P.); pasquale.capaccio@unimi.it (P.C.); 2 Division of Otorhinolaryngology, “San Maurizio” Hospital, 39100 Bolzano, Italy; luca.gazzini@sabes.it (L.G.); roberto.grigo@tiscali.it (R.G.); enrico.fazio@sabes.it (E.F.); monir.abousiam@sabes.it (M.A.); giovanni.giorgetti@sabes.it (G.G.); luca.calabrese@sabes.it (L.C.)

**Keywords:** head and neck reconstruction, periosteum, free flap, free periosteal flap

## Abstract

**Simple Summary:**

Head and neck bone reconstruction with revascularized free periosteal flaps and scaffold is an overlooked option in the literature. Aim of the present paper was to systematically analyse the results of maxillary and mandibular reconstruction with this technique. We found a total of 7 studies with 55 patients fitting with our inclusion criteria. The overall rate of complications was 43.7%. The success rate intended as scaffold integration resulted to be 74.5%. Our paper therefore highlighted that maxillary and mandibular reconstruction with revascularized free periosteal flaps and scaffold is a possible alternative in patient unable to bone free flap complex reconstruction, with a success rate higher to that of other secondary options.

**Abstract:**

Introduction: Head and neck bone reconstruction is a challenging surgical scenario. Although several strategies have been described in the literature, bone free flaps (BFFs) have become the preferred technique for large defects. Revascularized free periosteal flaps (FPFs) with support scaffold represents a possible alternative in compromised patient, BFF failure, or relapsing cancers as salvage treatment. However, only few clinical applications in head and neck are reported in literature. Purpose of the study was to systematically analyse the results of functional and oncologic maxillary and mandibular reconstruction with FPF with scaffold. Materials and Methods: A comprehensive review of the dedicated literature was performed according to the PRISMA guidelines searching on Scopus, PubMed/MEDLINE, Cochrane Library, Embase, Researchgate and Google Scholar databases using relevant keywords, phrases and medical subject headings (MeSH) terms. An excursus on the most valuable FPF’ harvesting sites was also carried out. Results: A total of 7 studies with 55 patients were included. Overall, the majority of the patients (*n* = 54, 98.1%) underwent an FPF reconstruction of the mandibular site. The most used technique was the radial forearm FPF with autologous frozen bone as scaffold (*n* = 40, 72.7%). The overall rate of complications was 43.7%. The success rate intended as scaffold integration resulted to be 74.5%. Conclusions: Maxillary and mandibular reconstruction with FPF and scaffold is a possible alternative in patient unfit for complex BFF reconstruction and it should be considered as a valid alternative in the sequential salvage surgery for locally advanced cancer. Moreover, it opens future scenarios in head and neck reconstructive surgery, as a promising tool that can be modelled to tailor complex 3D defects, with less morbidities to the donor site.

## 1. Introduction

Bone reconstruction in head and neck surgery is recognized as the most challenging, both for function of the recipient site and for morbidity to the donor site. Among the surgical possibilities, BFFs have become the preferred technique for reconstructions of maxillary and mandibular defects in adults and children [1]. When patient’s poor general conditions and comorbidities contraindicate the chance of a complex BFF reconstruction, alternative strategies with acceptable functional results are needed. The osteogenic capacity of periosteum is a well known property, exploited in various fields, from dentistry to orthopaedic trauma surgery and several experimental studies have clearly established the spontaneous or inducted bone regeneration from vascularized periosteum in animal models [2,3,4].

Revascularized FPF with support scaffold can represent a possible alternative in decision making algorithm for complex patients, BFF’s failure and disease recurrence, being more versatile than other less sophisticated choices such as pedicled flaps with reconstructive plates. However, only few clinical applications in head and neck reconstruction are reported in the literature [5,6,7,8,9,10,11].

To the best of our knowledge, no previous systematic review tried to summarize literature data on the use of FPFs in head and neck reconstruction. The aim of this paper was indeed to perform a qualitative analysis of the results of functional and oncologic maxillary and mandibular reconstruction with FPF with scaffold, with particular focus on immediate and late complications and on the success rate of the flap intended as integration and retainment of the support scaffold.

## 2. Materials and Methods

The study was performed according to the Preferred Reporting Items for Systematic Reviews and Meta-Analyzes (PRISMA) guidelines [12]. Since data were obtained from published literature, institutional review board approval and informed consent were not required for this study. No review protocol was registered for this study.

### 2.1. Eligibility Criteria

This systematic review was conducted according to PICOS acronym: Patients (P), patients underwent to head and neck bone reconstruction with FPF with scaffold; Intervention (I), head and neck bone reconstruction with FPF with scaffold; Comparator (C), observation; Outcomes (O), immediate complications, late complications, scaffold removal rate, success rate of reconstruction intended as integration of the support scaffold; Study design (S), retrospective cohort studies, case series, case report. Studies were excluded if they (a) were not in English, (b) were not available in full text form, (c) data were not extractable, (d) reported data of FPF reconstruction not involving the head and neck region, (e) reporting data of reconstruction with osteo-periosteal flap (i.e., medial femoral condyle osteo-periosteal free flap).

### 2.2. Data Source and Study Searching

A comprehensive review of the literature was performed on Scopus, PubMed/MEDLINE, Cochrane Library, Embase, Researchgate and Google Scholar databases using relevant keywords, phrases and MeSH terms. An example of a search strategy was the one used for PubMed/MEDLINE: “periosteal flap” [All Fields] AND (vascularized [MeSH Term] OR head and neck reconstruction [MeSH Terms] OR mandible, mandibular reconstruction [MeSH Terms]). The “cited by” function on Google Scholar was used to minimize the risk of missing relevant data. References were scrutinized for additional articles. The last search was carried out on 31 March 2021.

### 2.3. Data Extraction

Three independent reviewers (R.A., L.G. and L.N.) separately conducted the electronic search. All articles were initially screened for relevance by title and abstract, obtaining the full-text article if the abstract did not allow the investigators to assess the defined inclusion and exclusion criteria. Then, the authors independently assessed the full-text versions of each publication and excluded those whose content was judged not to be strictly related to the subject of this review. The conflict between reviewers was resolved by consensus. Data extraction from the included studies was systematically made using a structured form. If data were missing from the articles, then the corresponding author was contacted in an attempt to obtain the data.

## 3. Results

### 3.1. Literature Search Results

**Figure 1:**
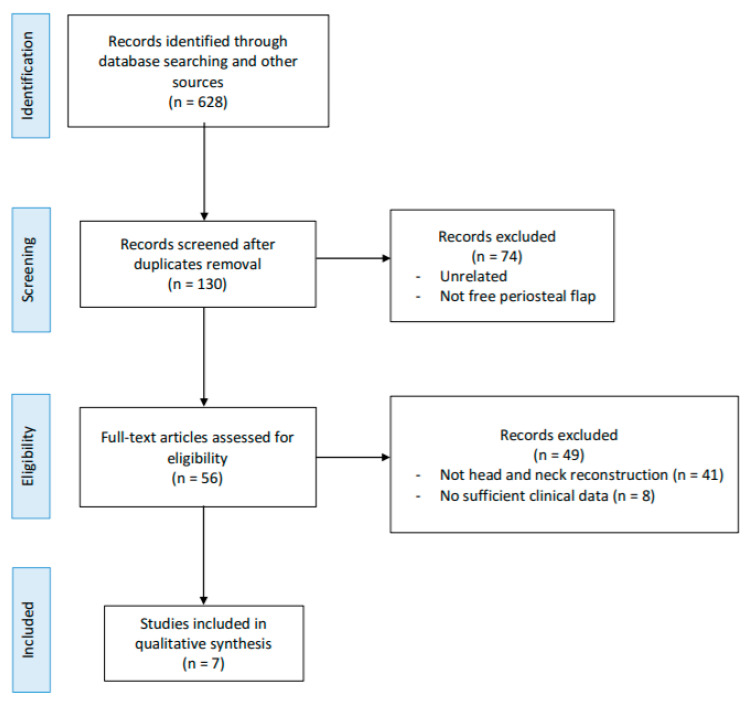
Preferred Reporting Items for Systematic Reviews and Meta-Analyzes 2009 flow diagram.

A flow chart of the study identification process is shown in Figure 1. A total of 628 citations were found searching in the selected databases after duplicates were removed. After title and abstract review, 572 articles were rejected and full texts of the remaining 56 papers were obtained and reviewed. After applying the inclusion and exclusion criteria, a total of seven studies were included in the qualitative synthesis (Table 1). The reasons behind the exclusions of 49 studies are shown in Figure 1. It was not possible to carry out a quantitative synthesis (meta-analysis) due to the studies’ design (mostly case report and small case series) and to the heterogeneity of the publications.

### 3.2. Patient Characteristics

A total of 55 patients were included in this review. All patients underwent maxillary or mandibular reconstruction using FPF with scaffold, the majority of the cases (*n* = 54, 98.1%) at the mandibular site. Forty-one (74.5%) patients underwent oncologic reconstruction, 13 (23.6%) were affected by osteoradionecrosis of the mandible and 1 (1.8%) underwent a post-traumatic reconstruction. With regards to defect type, 43 (78.2%) patients (41 with oncologic reconstruction and two with osteoradionecrosis) presented a segmental mandibular defect, 11 (20%) patients (all with osteoradionecrosis) needed a support mandibular reconstruction without resection and 1 (1.8%) patient presented a central midface post-traumatic loss of substance with a segmental maxillary reconstruction. The most used FPF resulted to be the radial forearm in 49 (89.1%) patients, while the preferred scaffold was the autologous frozen bone (*n* = 40, 72.7%).

Regarding the postoperative events, 25 (43.7%) patients experienced a complication, 21 (38.2%) immediate, treated with medical therapy or, alternatively, revision surgery and 3 (5.5%) a late complication, treated with revision surgery. As success rate, we have decided to consider the retainment and integration of the scaffold that is crucial for a good FPF reconstruction outcome: since 14 (25.5%) patients required scaffold removal both for immediate and late complications, the success rate resulted to be 74.5%.

## 4. Discussion

The FPF is a free flap that includes a periosteum paddle, which is harvested with the necessary support’s vessels able, once anastomosed at the recipient site, to revascularize the periosteum itself. The flap may include supporting surrounding tissue, such as the soft tissue of the skin paddle in the free forearm flap. This is a peculiarity that depends on the chosen donor site and which we will discuss in a subsequent section of the work.

This is the first systematic review trying to assess the role of FPFs in head and neck reconstruction. In particular, we tried to qualitative assess the immediate and late complications and the rate of success intended as integration of the support scaffold. In our opinion, this is the crucial point in a planned reconstruction with FPF. Indeed, despite the osteoinductive capacity of a revascularized FPF, in order to obtain a planned functional result, an adequate support able to guide the new ossification is mandatory; otherwise, the process would become afinalistic. Moreover, the aim of our analysis was to understand the role that FPFs with support scaffold can play in the algorithm for maxillary and mandibular reconstruction among the other options, such as BFFs or less sophisticated choices and as a salvage surgical treatment.

It is now appropriate to summarize the reasons why several authors have thought of exploiting FPF as a possible alternative in reconstructive surgery.

It is noteworthy that periosteum has a crucial role in bone regeneration and many authors over time tried to understand its biology and regenerative potential [2,3,4]. Historically, its osteogenic capacity was described for the first time in 1742 by Duhamel [13]. Ollier demonstrated that, upon treatment of bone fractures, integrity of periosteum must be retained to achieve successful healing [13,14]. Moreover, the author observed that transplanted periosteal tissue graft was capable of spontaneously inducing new bone growth and, a century later, also Finley et al. and Berggren et al., described this potential [15,16]. This remarkable capacity to induce bone growth and remodelling is related to the histologic characteristics of the periosteum. The tissue lines external surface of the bone, especially long bone and it is composed by two layers: the outer, which contains fibroblasts and the inner, source of skeletal progenitor cells and osteoblasts [3]. The latter being the most important in the process of bone regeneration [17]. Duchamp de Lageneste et al. showed that this efficient process depends on recruitment and activation of skeletal stem cells that allow cartilage and bone formation, leading to fracture consolidation [4,17]. Starting from these observations, several authors showed that a vascularized functional bone graft can be efficiently prefabricated with a vascularized periosteal flap and a support scaffold [18,19]. These assumptions are the basis for the in vivo bioreactor principle for producing vascularized functional bone, proposed since 2005 by Stevens et al. and Holt et al. [20,21]. The authors highlighted the advantages of periosteum compared to other tissues such as muscles. Indeed, periosteum is an ideal niche with skeletal progenitor/stem cells, abundance of molecular signals, increased nutrient-rich blood supply and prevent soft tissue infection [20]. Subsequently other studies confirmed the superior regenerative capacity of periosteum compared with alternative tissues [21,22,23,24,25,26,27,28,29]. It has been shown that intact periosteum is able to heal large defects in both long and flat bones [24], so different clinical applications have been observed [21,22,23,24,25,26,27,28,29,30,31]. In head and neck surgery some authors observed that preservation of periosteum in resective surgery, if oncologically safe, could lead to bone regeneration [32,33]. Ahmad and Omami described the abovementioned phenomenon in a mandibulectomy with periosteum preservation [33]. Zhang et al. reported a case of spontaneous bone regeneration after removal of free vascularised fibula flap hesitated in osteomyelitis [32]. They underlined that the periosteum of the fibula flap was left in place.

Several authors thought to take advantage of this property of the revascularized periosteum in the restoration of the bone loss of substance in post-traumatic fracture, chronic infections and oncological resections. The first applications regarded orthopaedic surgery [21,22,23,24,25,26,27,28,29]; subsequently, the attention was moved to maxillofacial district.

It is noteworthy that BFFs represent the gold standard in maxillary and mandibular reconstruction. The new cutting guide technologies allow to obtain a high profile functional and aesthetic result even in case of large mandibular resection or subtotal/radical maxillectomy [1]. For young patients with limited comorbidities this is the best chance for an efficient dental rehabilitation, an acceptable face profile and therefore, for a return to an almost normal social life after treatment. Unfortunately, there are some situations in which the use of a noble reconstruction may be contraindicated: (a) patients with serious comorbidities, (b) BFF failure (acute post-operative or as consequence of adjuvant treatment) and (c) relapsing disease involving the BFF [1,6,7]. The first is a condition that affects the choice of primary treatment, while the latter two affect the choice of sequential salvage treatment. Furthermore, it should be taken into consideration that the rehabilitation of the donor site, lower limb for fibula and iliac crest free flaps and upper limb for scapula free flap, is undoubtedly simpler and faster in a young and motivated patient [1].

Currently, secondary options differ site by site. Large maxillary reconstruction foresees as possible valid solutions obturator prostheses or, alternatively, temporalis muscle flap [34]. Instead, in case of mandibular resections, the classic salvage surgery involves the use of pedicle flaps such as pectoralis major muscle flap (PMMF) associated with reconstructive plates [35]. According to Dvorak et al., the use of PMMF with plate in head and neck can be even today considered the first choice for high-risk patients, free flap failure and salvage surgery [35]. These options can guarantee an immediate result in filling the lost substance. Nevertheless, although for an oldest old and/or complicated patient a maxillary reconstruction with prostheses or temporalis flap, or a mandibular restoration with pedicled flap and plate may be solutions with short operative timing, they certainly do not represent good choices for healthy elderly patients or for young patient who preferentially would need a definitive dental rehabilitation. Moreover, several authors reported a high rate of complications in reconstruction with PMMF and plate, up to 60% of cases, with almost 30% of major complications such as plate exposure and oro-cutaneous fistula, especially in previous irradiated patients or during adjuvant treatment [35,36,37].

The third option on the decision table is therefore the FPF. The idea of transferring a periosteal revascularized flap instead of a simple periosteal graft is based on the abovementioned observations, in particular by Stevens et al. and Holt et al. [20,21]. The revascularized periosteum, if supported by a valid scaffold, can generate reossification, which would make it suitable as a reconstruction tool in the maxilla and mandible, unless the patient presents absolute contraindications for a free flap. For these reasons, it is our opinion that FPF with scaffold should be considered as a routinely alternative to other second choices in decision-making algorithm. Usually, in head and neck reconstructive surgery, abnormal neo-ossification is considered a complication (e.g., ossification of the vascular pedicle of a free flap) [38]. However, as will be seen later, FPF may evolve in a guided vital bone segments when supported by an adequate scaffold, with better functional results rather than other options and with a possible ultimate dental rehabilitation. Kelley et al. firstly proposed the use of FPF in a case midface reconstruction, to optimize the vascularization of an autologous bone graft [5]. Afterwards, several authors proposed a more refined technique with FPF with autologous frozen bone for segmental mandibulectomy [6,7,8]. The first case series belong to Roselli and colleagues and Calabrese and colleagues [6,7]. Subsequently Cantù et al. reported a comparative experience on a group of 72 patients who had undergone to mandibular restoration with assorted techniques: among them, 33 underwent reconstruction with a fasciocutaneous forearm FPF associated with autologous frozen bone graft [8]. After mandibular resection, the technique described was based on detaching the soft tissues from the resected mandible, leaving on site the periosteum of the outer surface of the mandible, if oncologically safe. The bone segment was then frozen by immersion in liquid nitrogen for 10 min for a couple of times with a 20 minutes’ interval. The radial periosteum was used to wrap the inner surface of the replaced mandible, while the residual periosteum of the outer surface, if preserved, returned to its original place, over the outer surface of the mandible. In these experiences the surgical success was mild: indeed, while Roselli et al. and Calabrese et al., on very small series of cases reported a 100% of scaffold integration, despite some immediate and late complications, Cantù and colleague described an almost 40% of scaffold removal [6,7,8]. The most important complication was the breakdown of soft tissue suture with infection of the frozen bone graft. The authors did not noted differences between the use of a free flap with or without periosteum because the revitalization is long term procedure requiring several months [8], whereas the wound dehiscence normally occurs within a few days, when the bone is not yet revitalized. Moreover, postoperative radiotherapy increased the rate of failure, because it decreases the revascularization of the bone. A positive data reported by the authors was the less morbidity to the donor site and the shorter operative time, in case of FPF compared to a BFF [6,7,8]. Interestingly, the patients were investigated with technetium bone scan at 3 to 5 months after surgery, that showed a high rate of activity in the region of the reinserted mandibular segment, suggestive of the new ossification process. Cantù and colleagues concluded that, although the procedure is time-saving and cost-saving compared to BFF, this type of mandibular reconstruction must be reserved for patients with lateral tumours and severe comorbidities preventing more sophisticated bone reconstruction. Absolute contraindication must be the recurrence after radiotherapy. It is our opinion that this not impressive success rate was due to the quality of the scaffold rather than the FPF. In the literature we found only few experiences reported following that by Cantù et al. A single case experience was described in 2018 by Sierra et al. [9]. The authors used a bone allograft segment covered with a re-vascularized fibula periosteal flap in an 11-year-old patient with Ewing’s sarcoma of the mandible. The allograft used came from a cadaver tissue bank. According to Sierra and colleagues, the patient has showed optimal cosmetic, functional and radiological outcomes, without detecting donor-site complications in a long-term follow-up [9].

Bettoni and colleagues reported their experiences in managing osteoradionecrosis of the mandible with forearm fasciocutaneous FPF, showing acceptable results in patients without weakened mandibular arch [10]. A consistent difference with other experiences is that the authors used as “scaffold” the self osteoradionecrotic mandible of the patients. On 11 cases they reported 45.2% of immediate complications needing treatment, but a 90% of long-term success rate [10]. Moreover, postoperative radiograph showed evidence of new bone formation [10]. The authors underlined the efficiency to stop the development of osteoradionecrosis by providing periosteal bony vascular supplementation before the bony infrastructure weakens enough to require the use of a BFF [10]. The restoration of the bony tightness also avoids the use of inert osteosynthesis material, which in an irradiated area causes 6.5% to 57.7% of chronic complications [10]. A further statement that emerges from Bettoni and colleagues’ paper is that the composite FPF (fasciocutanous paddle and periosteal membrane) offers easy monitoring of the tissue viability and an additional interface which optimises the placement of FPF [10]. The skin paddle also allowed to correct oral scars.

Finally, it is of relevance a recent experience by Öhman et al. Purpose of the authors was to assess whether a customized titanium-hydroxyapatite bioceramic coated patient specific implants associated with soft tissue flaps can significantly contribute to the restoration of large mandibular defects [11]. Five consecutive patients with a general situation not suitable for BFF were operated for osteoradionecrosis with this technique [30]. The mean follow-up time was 12 months. It is important to underline that the reconstruction was not homogeneously carried out only with an FPF, but also with direct closure and PMMF. Two of the five cases were reconstructed with radial forearm FPF. The authors reported a stable long-term result in these 2 cases, with integration of the scaffold. In two other patients, one reconstructed with direct suture and 1 with PMMF, was necessary the removal of the implant [11].

The aforementioned experiences highlighted that, despite an efficient bone regeneration process from FPFs, the use of an inadequate scaffold can compromise the success of the reconstruction, due to early infections and rejection, bringing the success rate almost equal to that of reconstruction with PMMF and plate. However, overall, our analysis has demonstrated that FPF with scaffold had lower rate of scaffold rejection than a PMMF with plates reconstruction. The multiple scaffolds described in the literature are autologous bone graft from remote sites (e.g., iliac crest), autologous frozen bone from the surgical site, titanium-hydroxyapatite bioceramic coated implant and allograft demineralized bone matrix (DBM), a bone graft with the inorganic mineral removed, leaving the organic collagen matrix and native proteinaceous components [39]. The latter seems to be the most promising in experimental setting and in dental practice; nevertheless, it has never been used in head and neck reconstruction. DBM can be considered as scaffold rich in osteoinductive growth factors (e.g., bone morphogenetic proteins), sprouting capillaries, perivascular tissue and osteoprogenitor cells [39]. These growth factors modulate the differentiation of progenitor cells into osteoprogenitor cells, giving to DBM osteoconductive, osteoinductive and osteogenic properties, which are responsible for bone and cartilage formation [39]. There are various DBM on the market, such as Grafton^®^ and Osseograft^®^. Grafton^®^ was primarily studied as a bone graft extender for posterolateral spinal fusion in a rat model by Cammisa et al. in 2004 [39]. The authors compared Grafton^®^ and Osseograft^®^ with an in vitro study [39]. After an initial phase both Grafton^®^ and Osseograft^®^ induced an increased proliferative activity in the bone marrow stem cells, which reached a plateau after 10 days. These grafts also induced increased alkaline phosphatase activity of the osteoblast. Both are capable to induce osteoblastic proliferation and differentiation. Huang et al. proved the osteoinductive properties of DBM with rabbit skull periosteal flap pedicled on supraorbital artery as bioreactor demonstrating to obtain viable and properly modelable bone segments [40]. According to our review, the most used scaffold was the frozen autologous bone. The authors that used the frozen mandible wanted to return to the patient a support scaffold that could faithfully reproduced the removed section. The task of the FPF would have been to revitalize the reconstruction. However, the high rate of complication and scaffold removal suggested that this technique is not the best for ensuring integration of reconstruction. The other clinical data are currently too limited to define which is the ideal scaffold to support FPF. The experience reported by Öhman and colleagues seems optimistically promising since the two patients of the series reconstructed with the titanium coated implant had no complications with a 100% of scaffold integration [11]. In our opinion, custom made implants made of biocompatible material can represent an excellent compromise between function, being able to be adapted to the resected bone sections and integration with the FPF. It would be interesting to associate tailored biocompatible implants with DBM, in order to optimize the integration process.

In our review, none of the authors reported failures related to the free flap, nor were any advantages or disadvantages related to the harvesting site. The preferred site was the radial forearm, while the other sub-locations resulted anecdotal.

In the following section, we will examine the harvesting sites described in the literature.

### Surgical Options for Harvesting a Free Periosteal Flap

In the mid-1980s, Van den Wildenberg et al., in a study on animal models, demonstrated that the osteogenic capacity of vascularized periosteum varies between different donor sites. They concluded that long bone periosteum should be more efficient [41]. Subsequently, in the early 1990s, human anatomic studies were carried out with the aim to identify all the possible sites for FPFs harvesting [42]. In the literature, we found that, so far, in head and neck reconstruction the FPFs actually used were radial forearm, internal femoral condyle, fibula and humerus [6,7,8,9,10,11]. However, other sites were described. According to Penteado et al., there are six possible harvesting sites: humerus, radius, ulna, iliac fossa, femur and tibia [42]. Penteado and colleagues. Performed a cadaveric study on 25 fresh specimens injected with coloured latex. The aim was to check all the potential donor sites supplied by a constant vascular pedicle with vessels of wide calibre, sufficient to allow an adequate dissection of the pedicle and subsequent microvascular anastomosis [42]. The authors concluded that the more suitable donor sites among the 6 described should be: distal third of the humerus, iliac crest and distal third of the femur [42].

Starting from the upper limb, elective humerus periosteum donor site was supposed to be the lateral and anterior aspect of the distal half of the bone, supplied by the posterior branch of the deep brachial artery [42].

The use of radial forearm FPF in head and neck reconstruction was reported by several authors, both for oncological reconstruction, post-traumatic reconstruction and for the management of the osteoradionecrosis [11,43]. This flap resulted the favourite in our review, having been used in almost 90% of the patient included. It has the advantage of being easier to harvest than deeper sites such as fibula or iliac crest, causing less morbidity to the patient. It can also be easily prepared with a fasciocutaneous paddle, very useful for the restoration of the soft tissues lost during the resection phase and for monitoring the flap in the postoperative setting. It is supplied in the proximal fourth by branches of the common interosseus artery and in the whole distal part by the periosteal branches of the anterior interosseus artery. Bettoni et al. described in detail the harvesting technique [43]. The authors underlined the importance to dissect the fasciocutaneous paddle by the radial side of the flap: the radial periosteum should be incised with the length corresponding to the slice of bone section to be resurfaced and gently dissected to avoid dissociation from radial pedicle [43].

Periosteum of the medial surface of the iliac crest is feed by branches of the deep circumflex iliac artery. On this site, Penteado et al. suggested to harvest a muscular-periosteal flap including the iliac muscle in order to preserve the periosteal vascularization [42].

Periosteum from femoral condyle was the donor site that showed a wide development in clinical practice [44,45,46,47]. The lower third of the femur has a double vascularization: a branch of the femoral artery and the lateral metaphyseal artery, arising from the descending genicular artery. The second generates a rich vascular anastomotic network with the innominate branch at the anterior aspect of the femur. The latter is the elective vascular supply of the medial femur condyle free flap, because of the longer length of the pedicle, ideal for microvascular anastomosis [44,45,46,47]. Regarding the development of the aforementioned donor flap in head and neck, Choi et al. reported a case of reconstruction of median facial dysplasia using the medial condyle osteo-periosteal free flap [45], while Enzinger et al. reported a mandibular condyle reconstruction with a lateral condyle osteo-periosteal free flap [47].

Crock and colleagues carried out an anatomical study describing the vascularization pattern of the tibial periosteum [48]. The periosteal branches arise from the posteromedial portion of the anterior tibial artery approximately every 3 cm, they run upon the interosseus membrane accompanied by paired concomitant veins and, finally, converge in rings around the shaft of the tibia feeding the periosteum. Parallel rings are connected by anastomotic vessels running in a longitudinal way. Moreover, the authors described a case report on a fracture of the tibial midshaft successfully repaired with a pedicled periosteal flap harvested from the lateral surface of the middle third of the tibial shaft based on the aforementioned anterior tibial artery vascular pedicle [48]. However, this is not considered at the moment a suitable donor site for FPF, because the sacrifice of the anterior tibial artery could prejudice the blood supply of the muscles of the anterior compartment [48].

Fibula has a strong segmental vascularization which is given by the peroneal artery, one of the three branches terminalis of popliteal artery, together with tibialis anterior and posterior arteries [9]. The peroneal artery with its two comitant veins derived, together with the posterior tibial artery, from the popliteal artery about 7 cm below the knee and runs parallel along the medial axis of the fibula, leaving numerous segmental periosteal vessels, that are the basis of bone and periosteum supply. The use of fibula FPF for mandibular reconstruction was reported by Sierra et al. [9].

## 5. Conclusions

Head and neck bone reconstruction with FPF and scaffold is a possible and intriguing alternative to other options in the salvage surgery setting. It should be considered a valid choice instead of simple reconstruction with pedicled flap and plate, as it can offer a more functional and aesthetic result, especially in young and healthy patients, where there has been failure of BFF or relapse of disease. Moreover, FPFs open possible future scenarios in maxillary and mandibular reconstruction, as promising tool that can be modelled to tailor complex three-dimensional defects, with less morbidities to the donor site.

Our review of the literature showed that, up to now, FPFs were used only occasionally in maxillary and mandibular surgery, with, even if not optimal, at least good results in terms of success rate in comparison with other less sophisticated reconstructive options. Furthermore, the available papers are mostly case reports and small case series and this represents the major weakness of our study.

In our opinion, larger studies are needed to understand the role that association of FPF with custom made biocompatible implants and DBM could actually have in the future of head and neck reconstructive surgery.

## Figures and Tables

**Table 1:** Characteristics of included studies.

Study (Year)	Study Design	Site of Reconstruction (Reason)	N° of FPF Reconstruction	Type of FPF (N°)	TYPE OF SCAFFOLD	Immediate Complications (%)	Late Complications (%)	Scaffold Removed (%)	Rate of Success— Scaffold Integrated (%)
Kelley (2003)	CR	maxilla (post-traumatic)	1/1	radial forearm (1)	iliac crest autograft	none (0)	none (0)	none (0)	1 (100)
Roselli (2004)	CS	mandible (oncologic)	2/2	radial forearm (2)	autologous frozen bone	none (0)	none (0)	none (0)	2 (100)
Calabrese (2007)	R	mandible (oncologic)	5/7	radial forearm (5)	autologous frozen bone	subacute infection (60)	partial sequestrum (60)	none (0)	5 (100)
Cantù (2009)	R	mandible (oncologic)	33/72	radial forearm (33)	autologous frozen bone	suture dehiscence (39.4)	none (0)	13/33 (39.4)	20/33 (60.6)
Sierra (2018)	CR	mandible (oncologic)	1/1	fibula (1)	bone cadaver allograft	none (0)	none (0)	none (0)	1 (100)
Bettoni (2019)	R	mandible (radionecrosis)	11/11	radial forearm (6) internal femoral condyle (4) external brachial—humeral (1)	self radionecrotic mandible	total (45.2) - necrosis (18.2) - fracture (9) - fistula (9) - hematoma (9)	none (0)	1 (9.9)	10 (90.1)
Öhman (2019)	CS	mandible (radionecrosis)	2/5	radial forearm (2)	titanium-hydroxyapatite bioceramic coated implant	none (0)	none (0)	none (0)	2 (100)

Abbreviations: FPF, free periosteal flap; CR, case report; CS, case series; R, review.

## Data Availability

The data presented in this study are available on request from the corresponding author.
